# Efficient detection and assembly of non-reference DNA sequences with synthetic long reads

**DOI:** 10.1093/nar/gkac653

**Published:** 2022-08-04

**Authors:** Dmitry Meleshko, Rui Yang, Patrick Marks, Stephen Williams, Iman Hajirasouliha

**Affiliations:** Tri-Institutional PhD Program in Computational Biology and Medicine, Weill Cornell Medical College, NY 10021, USA; Institute for Computational Biomedicine, Department of Physiology and Biophysics, Weill Cornell Medicine of Cornell University, NY 10021, USA; Tri-Institutional PhD Program in Computational Biology and Medicine, Weill Cornell Medical College, NY 10021, USA; Institute for Computational Biomedicine, Department of Physiology and Biophysics, Weill Cornell Medicine of Cornell University, NY 10021, USA; 10x Genomics Inc., Stoneridge Mall Road, Pleasanton, CA 94566, USA; 10x Genomics Inc., Stoneridge Mall Road, Pleasanton, CA 94566, USA; Institute for Computational Biomedicine, Department of Physiology and Biophysics, Weill Cornell Medicine of Cornell University, NY 10021, USA; Englander Institute for Precision Medicine, The Meyer Cancer Center, Weill Cornell Medicine, NY 10021, USA

## Abstract

Recent pan-genome studies have revealed an abundance of DNA sequences in human genomes that are not present in the reference genome. A lion’s share of these non-reference sequences (NRSs) cannot be reliably assembled or placed on the reference genome. Improvements in long-read and synthetic long-read (aka linked-read) technologies have great potential for the characterization of NRSs. While synthetic long reads require less input DNA than long-read datasets, they are algorithmically more challenging to use. Except for computationally expensive whole-genome assembly methods, there is no synthetic long-read method for NRS detection. We propose a novel integrated alignment-based and local assembly-based algorithm, Novel-X, that uses the barcode information encoded in synthetic long reads to improve the detection of such events without a whole-genome *de novo* assembly. Our evaluations demonstrate that Novel-X finds many non-reference sequences that cannot be found by state-of-the-art short-read methods. We applied Novel-X to a diverse set of 68 samples from the Polaris HiSeq 4000 PGx cohort. Novel-X discovered 16 691 NRS insertions of size > 300 bp (total length 18.2 Mb). Many of them are population specific or may have a functional impact.

## INTRODUCTION

Long-read sequencing technologies such as PacBio and Oxford Nanopore are able to improve genome-wide structural variant (SV) characterization and *de novo* assembly (e.g. (1–4)). Both PacBio and Oxford Nanopore require high input DNA amounts (at least 100 ng but ideally micrograms of DNA). For some real-world applications, the high input requirements limit the use of long-read protocols. In particular, for the whole-genome sequencing of clinical tumor samples, we may not have enough DNA material to leverage long-read sequencing.

Low-cost, low-input (i.e. only 1–5 ng) and highly accurate synthetic long-read technologies such as the 10x Genomics system (5), BGI Long Fragment Reads (stLFR) (6) and Universal Sequencing Technology (UST TELL-Seq) (7) have recently emerged to improve the ability of standard short-read sequencing technologies in characterizing whole genomes and metagenomes. In synthetic long-read sequencing, DNA molecules are sheared into long fragments (i.e. 10 to a few hundred thousand base pairs), and barcoded short reads from these long fragments are produced in such a way that reads from a long fragment share the same barcode. Such reads are referred to as linked-reads and the barcodes provide additional long-range linkage information about the target genome being sequenced. For more details of the synthetic long-read sequencing process and related protocols, we refer the reader to (5). 10x Genomics introduced one of the first commercial platforms for synthetic long-read sequencing and gained popularity in just three short years. Thousands of whole genomes have recently been sequenced using 10x Genomics linked-reads including the InPSYght Consortium which has sequenced a schizophrenia case/control cohort of 545 individuals. While 10x Genomics has since discontinued (as of June 30, 2020) shipment of their kits due to patent issues with Bio-Rad Laboratories, several other technologies such as Single Tube Long Fragment Read (stLFR) from BGI-MGI and Transposase Enzyme Linked Long-read Sequencing (TELL-Seq) from Universal Sequencing have recently made similar synthetic long-read sequencing platforms commercially available. Indeed the single-tube approach for generating synthetic long reads in stLFR and TELL-Seq eliminates the need for a 10x Chromium-like instrument. This made the cost of synthetic long-read sequencing even cheaper than 10x Chromium linked-reads and only slightly more than the typical Illumina cost (6,7).

Synthetic long reads have proven to be useful in multiple applications including but not limited to genome assembly (8,9), genome phasing (5,10), metagenomics (7,11–13), or large-scale SV detection or general novel adjacencies (14–18). The contribution of synthetic long reads to SV detection is, however, still limited to large structural variations (i.e. at least several thousand base pairs) and only certain classes of SVs, because the techniques for detecting SVs using synthetic long reads mainly rely on quantifying barcode similarity of aligned reads between distant pairs of genomic locations to identify novel adjacencies in a sequenced genome. Therefore, virtually none of the available SV detection algorithms attempts to characterize and assemble non-reference sequences, i.e. DNA sequences present in a given sequenced sample but missing in the reference genome. The latest attempts at resolving the full spectrum of genome variations using synthetic long reads does not include insertions (7,19), and less sensitive whole-genome *de novo* assembly methods for this purpose (9,20,21) are computationally expensive.

Non-reference sequences (NRSs) refer to new DNA sequences that are missing in the reference genome but present in a sequenced sample. This type of insertion is distinct from repeat insertions such as mobile element insertions. NRSs may contain functional elements and are of great interest for human genome diversity (22). Without accurate detection and precise placement of these NRSs, it is impossible to build new human reference genomes and correct misassemblies in existing references. Despite several attempts in recent years, insertion events remain poorly characterized because of the limitations of short-read and synthetic long-read technologies. The main focus of large-scale whole-genome consortium projects (e.g. the 1000 Genomes Project) was to detect deletions. However, pan-genome assembly projects show a substantially large number of NRS insertions in sequenced human genomes not present in the reference genome (22–24). In particular, a recent paper describing the assembly of a pan-genome from deep sequencing of 910 humans of African descent (22) claimed that close to 300 Mbp of DNA sequence in 125 715 distinct contigs are missing from the reference genome but are present in the pan-genome. The vast majority of these contigs, however, could not be placed on the reference genome because of the limitations of short reads. Only 1548 (total length 4.4 Mb) of the contigs were placed on the reference genome, which is < 1.5% of the new sequences found.

Several approaches attempted NRS detection using standard short-read sequencing without whole-genome *de novo* assembly. For example, NovelSeq (25) was developed to characterize these insertions using high coverage ultra short-read datasets (reads of length 35–41 bp). This algorithm was applied to the high coverage samples in the 1000 Genomes pilot phase, and a total of 128 NovelSeq calls were reported and validated (26, 27). Subsequent short-read methods for this problem such as MindTheGap (28), ANISE and BASIL (29), PopIns2 (30) or Pamir (31) appended population-based techniques (e.g. pooling multiple low-coverage samples from the same population) or used additional whole-genome signals such as split reads for better breakpoint resolution. All of these short-read methods are based on the idea of assembling reads that are not aligned to the reference genome and connecting these assembled sequences with potential insertion breakpoints on the reference genome using paired-end information. NovelSeq (25) was the first algorithm that capitalized on this idea. NovelSeq identifies unaligned paired-end reads with a single-end read aligned (i.e. One-End-Anchor reads) and performs a local assembly of those One-End-Anchor reads that clustered around the same positions on the reference. Sequence contigs assembled in this way are simply called ‘anchors’ and we use this term throughout this manuscript as well. An anchor represents a piece of sequence that can be located on the reference genome and can be used to find the breakpoint of a potential NRS insertion. NovelSeq then uses a *de novo* assembler such as ABySS (32) to assemble those reads where none of their ends mapped to the reference (called orphan reads) and finally merges assembled contigs from the orphan reads with anchors using a greedy matching algorithm. MindTheGap (28) uses a novel *k*-mer-based signature to find insertion sites on the reference genome, while ANISE (29) employs a new algorithm for resolving certain repeat copies. PopIns2 (30) is an alternative algorithm that uses information from several whole-genome samples to find NRS insertions common to an ancestral population. Pamir (31) also generalizes NovelSeq’s approach for handling several low-coverage genomes from the same population. Moreover, the accuracy and number of NRS insertion discoveries were improved due to split-read signature usage and a non-greedy approach to match NRSs with their anchors.

All of the existing short-read methods fail to correctly assemble and locate the vast majority of insertions that are > 300 bp. The sensitivity of short-read methods is limited to smaller insertions. Also, short-read libraries dramatically reduce our ability to locate an inserted sequence on the reference genome because the size of anchors is limited by the small insert size of the library and is usually less than the size of common repetitive elements. In light of these challenges, our main contribution here is a novel technique that can leverage barcodes and long fragment information encoded in synthetic long-read sequencing to achieve much longer anchors. Our technique allows determination of the unambiguous location of non-reference sequences on the reference even inside certain repetitive regions, a major limitation of short-read methods (see Supplementary Figure S1 for a demonstration).

Indeed it is possible to leverage *de novo* assembly of synthetic long reads for NRS detection. For example, NUI (20) calls NRS insertions specifically using synthetic long-read data. It assembles the whole dataset with SuperNova (9), aligns poorly aligned reads into assembled contigs to identify insertion sequences and then aligns contigs with an insertion to the reference genome to find the position of breakpoints. However, whole-genome assembly is a computationally expensive task. Additionally, assembly of repetitive regions is extremely challenging, and insertions in those regions are likely to be overlooked due to fragmentation. In this study, we introduce an integrated mapping-based and assembly-based algorithm using synthetic long reads, which is substantially more accurate than existing methods for NRS insertion discovery and placing the insertions on the reference genome. In addition, our method is computationally more efficient compared with those synthetic long-read approaches that use whole-genome *de novo* assembly such as (9,20) because we only process a very small fraction of informative linked-reads and not the whole target genome sequencing data. Our synthetic long-read method is able to characterize one of the most challenging classes of SVs with a reasonable additional cost to standard short-read sequencing. Here we evaluated our method on several cohorts of synthetic long reads sequenced on 10x Genomics, TELL-Seq and stLFR platforms. Our method can also be applied to other types of synthetic long-read data when they become widely available.

Our method can especially handle insertions longer than 300 bp, a major limitation of existing methods, and we show that our method is highly sensitive when tested on both simulated data and real data including several well-characterized haploid and diploid human genomes. We also present the findings of our method when applied on 68 human whole-genome samples as part of the Polaris PGx cohort.

## MATERIALS AND METHODS

In this section, we describe our method, Novel-X, for detection of NRSs using synthetic long-read sequencing. This method is based on a novel idea that the barcode information encoded in synthetic long reads can be used to reconstruct long anchors that can be unambiguously placed on the reference genome. This allows us to find exact breakpoint positions on the reference even in certain repeat regions. Our approach is based on the local assembly of multiple barcodes originating from the same genomic loci.

The input for Novel-X is a reference genome (e.g. GRCh38) and a BAM file produced by aligning barcoded linked-reads to the reference genome. For aligning 10x Genomics barcoded reads, the standard LongRanger package (https://support.10xgenomics.com/genome-exome/software/downloads/latest) or EMA (33) can be used. We refer to the set of the reads from the input BAM file as original reads and to the BAM file itself as original BAM. A pre-processing step in Novel-X is the extraction of paired-end reads from the original BAM that cannot be aligned to the reference genomes or have poor alignments. Intuitively, NRSs should consist of reads that do not align anywhere on the reference. The number of such insertions is typically not high. Therefore, in contrast to whole-genome *de novo* assembly methods, we filter out a majority of reads which have high-quality alignments to make the pipeline computationally effective. We choose paired-end reads with at least one end not aligned to the reference genome, or aligned but with a mapping quality below 10, or that have > 20% of soft-clipped bases. We believe that such reads are likely to contain a large portion of insertion sequences, and therefore are important for identification of NRS insertions. Additionally, we choose only reads with the average base quality score > 20. We collectively call this set of unaligned reads $\mathcal {U}$. Reads from $\mathcal {U}$ correspond to NRS insertions and contain parts of anchor sequences.

Novel-X is comprised of four steps, taking as input the reads in $\mathcal {U}$ and generating as output a list of candidate NRS sequences. (i) **NRS insertion assembly**—assembly of all reads in $\mathcal {U}$. As the result of this step we obtain a set of NRS candidates. (ii) **Barcode list extraction**—for each insertion candidate we find barcodes with at least one read aligned to this insertion. (iii) **Insertions reassembly**—we reassemble reads with barcodes found in the previous step to obtain anchors for each insertion. (iv) **Location of insertions on the reference—**we locate these anchors on the reference genome and find the exact position of each insertion.

**Figure 1.**
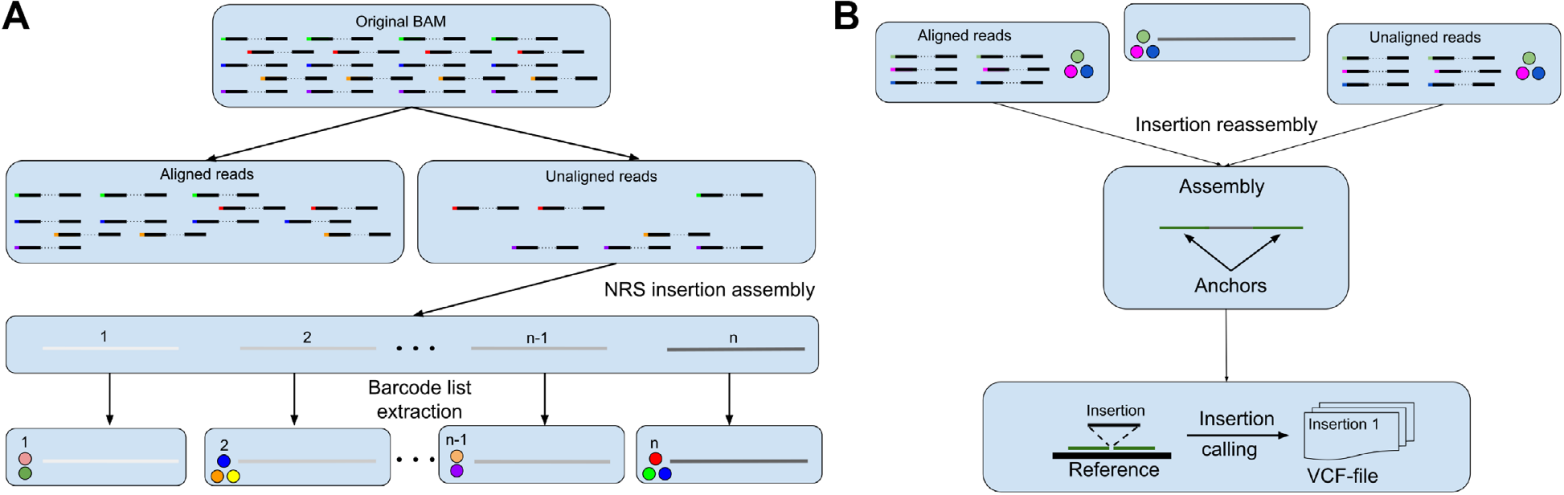
Novel-X outline. (**A**) Schematic original BAM filtering, NRS insertion assembly and barcode read extraction steps. At the end of these steps, we get *n* contigs with the barcode lists. (**B**) For each contig from the previous step, we pool aligned and unaligned reads, assemble them and call insertions from the reassembled contigs.

We describe each step in detail below. An overview of our technique is also shown in Figure 1.

### NRS insertion assembly

We first use the Velvet assembler (34) to assemble reads in $\mathcal {U}$. Velvet is an assembler of choice for our purpose because other assemblers often have specific assumptions about the data such as diploidy or contiguity of a fully sequenced genome. In our experiments, other assemblers produce worse results on the simulated data (see Supplementary Table S2). Note that, *de novo* assembly of all reads in a high-coverage whole-genome sample is a computationally expensive task. However, $\mathcal {U}$ consists of a small fraction of the total reads (usually 2–3%) and can be assembled efficiently. Ideally, the resulting assembly contigs would belong to sequences of novel insertions but could also be the results of misassembly or originate from non-human contaminant sequences. If needed, we can perform a contamination removal procedure similar to what was previously done in Pamir (31), i.e. perform a BLAST search against an nt/nr database and filter out all contigs that align to non-human references. We implemented this contamination removal procedure as an option in our software because it may also remove some known viral sequences (e.g. human herpesvirus sequence) that can be of interest for certain users. Similar to the analogy in (25), we call the remaining contigs orphan contigs.

### Barcode list extraction

For each orphan contig *c*, we first align the reads in $\mathcal {U}$ to *c* and filter read alignments with low-quality scores or with a large fraction (≥ 20%) of soft-clipped or hard-clipped sequences. Note that the exact definition of soft- and hard-clipped reads is aligner specific. Intuitively, soft-clipped and hard-clipped read parts represent the part of a read that cannot be aligned together with the remainder of the read due to a lack of sequence similarity. Let *R*(*c*) be the set of filtered barcoded reads aligned to *c*. We denote *B*(*c*) as the set of all barcodes in *R*(*c*). We extract and store every read from the set of original reads whose barcode is in *B*(*c*). The information about barcodes of remaining reads is, however, extracted and aggregated separately for each orphan contig. Each contig that recruits less than *t* barcodes is discarded since the joint assembly of a limited number of barcodes is very unlikely to produce long anchors during the next step of the algorithm. In our software, we set the parameter *t* to 5 as default, but this parameter can also be defined by the user.

### Insertion reassembly

**Figure 2.**
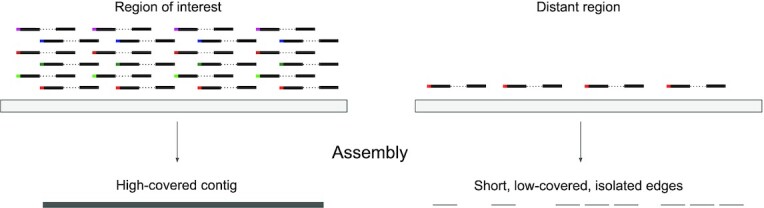
Underlying long fragment alignment to regions of interest (i.e. novel insertion site) and some distant genomic region and subsequent assembly. Every barcode extracted from the barcode list recruits some underlying long molecule aligning to the insertion site. Long molecules with the same barcode can be recruited to align to the offsite genomic region but the probability of recruiting multiple long molecules in the same bin is small and assembly of such regions into contiguous sequences is unlikely.

In order to assemble NRSs together with their anchors, for each barcode list *B*(*c*) we search the original BAM and extract the reads that have a barcode from *B*(*c*). Both aligned and unaligned reads are extracted during this stage. Then, we reassemble extracted reads for each *B*(*c*) separately. By default, we use SPAdes (35) with *k*=77 (that empirically works better than other standard single *k*-mer lengths) and cov-cutoff = 3 (to remove low-covered spurious sequences), though usage of other assembler options might also be feasible. One confounding factor is that multiple genomic fragments can share the same barcode, which means that reads from different loci might be inappropriately recruited to assemble NRS anchors. However, because of lower coverage of recruited reads in regions other than NRS anchors, SPAdes interprets these inappropriately recruited reads as sequencing errors or contamination, and edges made from these reads will be deleted from the assembly graph (see Figure 2).

In order to support this claim, we create a simple model and calculate the probability of the assembly of a random region under this model. Based on given parameters associated with a synthetic long-read experiment, we can indeed model the genome as a set of non-intersecting regions and calculate the probability of aligning underlying long fragments in regions of no interest. In Supplementary text A: ‘Proof: A random region has a low coverage’, we combinatorially proved that the probability of getting a significant number of long fragments in a given region is small and its assembly is unlikely.

After the reassembly step, we only perform downstream analyses of reassembled contigs that have a best match with an existing orphan contig. This match is found using Minimap2 (36) alignment. Ideally, the result can be presented as a single contig with ‘left anchor–insertion sequence–right anchor’ structure.

### Location of insertions on the reference

The last step of our pipeline is finding the positions on the reference genome where the novel insertions can be placed. We use Minimap2 for aligning the resulting contigs from the previous step to the reference genome. It allows us to align any number of candidate sequences to the human genome in a reasonable time with high accuracy. Since Minimap2 results contain a large portion of short spurious alignments, we use a filtering procedure that resembles the QUAST (37) procedure, i.e. choosing best alignment subsets from a given alignment set that maximizes the number of continuously covered base pairs. The remaining alignments that are adjacent with respect to the reference genome are analyzed for insertion signatures. If the distance between adjacent anchor alignments is small on the reference but large on the contig, then the contig probably contains an NRS. Otherwise, the contig is discarded. The insertion content can be found as a subsequence on the contig between these alignments. We only output insertions longer than 300 bp with the sum of the anchors’ lengths exceeding 300 bp to prevent false calls. All identified insertions are stored in a vcf (Variant Call Format) file. Placed insertions that do not satisfy the conditions above (e.g. smaller insertions) are stored in a separate file as described in our documentation.

## RESULTS

### Evaluation on simulated data

We first developed a simulation scenario to evaluate Novel-X in a fully controlled setting. We inserted 2000 randomly generated DNA sequences between 50 and 6000 bp in length into the GRCh38 reference genome. Synthetic long reads were simulated using LRSIM (38) with 65× sequence coverage. We evaluated Novel-X, PopIns2 (30), NUI (20) and Pamir (31) on these data. Because Pamir is not able to work directly with synthetic long-read data, we simulated matching standard short-read data of the same coverage from the same genome using ART (39). We also tried Manta (40) as a popular tool for the general short-read data SV calling. However, Manta was not able to detect any novel insertions longer than 300 bp.

For the purpose of comparison, we consider a pair of insertions called by two different methods the same event if their breakpoint positions on the reference genome differ by no more than 100 bp and their length differs by no more than 5%. Table 1 shows the number of NRS insertions found by different methods and their sizes. Our simulations demonstrate that Novel-X has a high recall and precision, especially for longer insertions which are more challenging to detect. While NUI’s results were comparable with those of Novel-X, PopIns2 had a lower recall. Pamir had the lowest recall overall and had far from perfect precision, but achieved the highest recall for insertions from the 50–299 bin. Novel-X achieved the almost perfect precision of 0.999, NUI 0.997, PopIns2 0.999 and Pamir 0.817. Overall, Novel-X has the best performance, while NUI has the second best. We checked if the sequence content of the predicted insertions matched the ground truth sequences by using a global sequence alignment and a nucleotide identity threshold of 80%. All sequence contents of Novel-X, PopIns2 and Pamir insertions matched, and NUI produced only 48 incorrect sequences (< 3%).

**Table 1.** Length breakdown of insertions found by different methods in the simulated dataset

Length (bp)	50–299	300–499	500–999	1000–1999	2000–4999	≥5000	Total TP	FP
Total	83	73	148	361	1023	312	2000	
Novel-X	47	**68**	**137**	**336**	**928**	**273**	**1789**	**1**
PopIns2	3	60	124	299	860	242	1588	**1**
Pamir	**76**	56	99	213	616	179	1239	278
NUI	73	60	131	313	902	271	1750	3

Novel-X found a majority of insertions longer than 300 bp and had the best performance.

#### Low-coverage genomes

To show the utility of our method on low-coverage synthetic long-read genomes, we also studied the effect of downsampling the sequencing data on Novel-X performance. From the original 65× coverage, we downsampled the simulated dataset with the ratios 0.8 (52×), 0.6 (39×), 0.4 (26×) and 0.2 (13×). We evaluated Novel-X, PopIns2 and NUI performances on these datasets (see Supplementary Table S1). NUI and Novel-X have a stable performance with 26× coverage and above. The performance only dropped when we tested it with the 13× sequence coverage. In that case, the very low coverage prevented the assembly of long anchors and insertion placement. PopIns2 seems to have the highest requirement for data coverage, because its performance worsens gradually with every downsample step.

#### Choice of assemblers

The Novel-X method uses different assemblers for different tasks. Using simulated data, we were able to confirm that Velvet v1.2.10 (34) for the assembly of unaligned reads, and SPAdes v3.15 (35) for insertion reassembly, are the best practice (see Supplementary Table S2). SuperNova is not suitable for the insertion reassembly step, because it has a prohibitive running time. Each SuperNova run requires minutes even with a small subsample of only a few thousands reads. Velvet and SPAdes also provide a reasonable running time.

### Evaluation on real datasets

**Figure 3.**
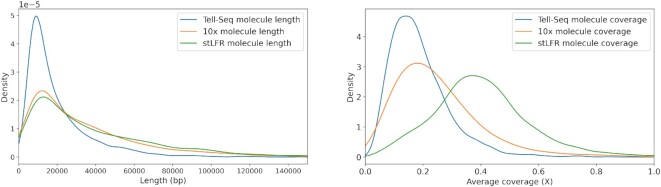
Synthetic long-read properties of different platforms on the NA12878 dataset are shown. Long molecule length (left) is the distance between the leftmost and the rightmost alignment of the reads with the same barcode within the same genomic region. Long molecule coverage (right) is the coverage of the genomic region by the reads with the same barcode.

In order to evaluate the utility of Novel-X on real data, we performed experiments with several synthetic long-read datasets from different platforms (10x, Tell-Seq and stLFR). We observed that different synthetic long-read platforms have different properties. In particular, the average long molecule length, the number of barcodes or the average coverage with short reads differ from one platform to another. Figure 3 shows a comparison of the molecule length distribution and the molecule coverage distribution of different synthetic long-read platforms on the NA12878 genome. stLFR and 10x platforms have almost identical molecule length distribution with a higher density of longer molecules compared with Tell-Seq. The molecule coverage distribution has distinct patterns for each technology. The majority of Tell-Seq molecules have coverage below 0.3×. The peaks of molecule coverage distribution for 10x and Tell-Seq have similar values, but the 10x molecule coverage distribution has a higher density tail. The peak of stLFR’s molecule coverage distribution is very high, ∼0.4×. In stLFR and 10x, some of the molecules have a coverage of > 1. The maximum coverage of a molecule for stLFR is 1.6× and for 10x is 7.6×.

In total, we used nine synthetic long-read whole-genome datasets in our experiments with real data (Table 2). These datasets were chosen because high coverage long-read datasets and/or integrated benchmark callsets (example from the Genome in a Bottle study (41)) were available as ground-truth callsets.

**Table 2.** Overview of real synthetic long-read datasets used for benchmarking in this study

Dataset		Number of	Reference	Validation
(Platform)	Coverage	barcodes	genome	set
HG002 10x	59×	3956724	GRCh37	(41)
HG002 TELL-Seq	42×	10543778	GRCh37	(41)
HG002 stLFR	35×	4653424	GRCh37	(41)
NA12878 10x	60×	3956724	GRCh38	(42)
NA12878 TELL-Seq	41×	15198586	GRCh38	(42)
NA12878 stLFR	40×	3962939	GRCh38	(42)
NA19240 10x	73×	4155776	GRCh38	(43)
CHM1 10x	40×	2758147	GRCh38	(44)
CHM13 10x	40×	2701721	GRCh38	(44)

LongRanger is the standard aligner for synthetic long-read platforms. TELL-Seq datasets can be aligned to the reference genome using LongRanger with an altered barcode list file (7). stLFR datasets can also be converted to a LongRanger-compatible format using the stlfr2supernova pipeline. After these steps, LongRanger BAM files can be used in the Novel-X pipeline directly.

We aimed to run every tool on every dataset, but we were faced with certain limitations. NUI-pipeline and SuperNova/Paftools require whole-genome assembly made by SuperNova, which is a diploid assembler. Therefore, they do not work with our CHM1 or CHM13 haploid datasets. The short-read tool Pamir is not designed to handle sequencing data in which the two ends of paired-end reads have different lengths. This is because the mrsFast aligner used inside Pamir requires both ends to be of the same length. In Tell-Seq and 10x data, the ends often have different lengths, so only stLFR technology provides paired reads with equal length ends. In order to run Pamir on additional datasets, and match the synthetic long-read dataset coverage for CHM1 and CHM13 genomes (50×) to WGS dataset coverage, we downsampled the synthetic long-read datasets at a rate of 0.8, therefore all data that we use have 40× coverage. After examining Pamir’s results on these datasets, we decided not to match NA19240, NA12878 and HG002 datasets because results were much worse than Novel-X, NUI and SuperNova for all processed datasets.

In order to validate calls, we matched them against corresponding long-read and the GIAB integrated SV callsets, that were previously published (see Table 2 for references). These callsets include all types of SVs, while Novel-X, PopIns2, NUI and Pamir call only unique NRS insertions. In order to filter other insertion calls such as repeat insertions from the SMRT-SV callset, for each sample, we aligned the unaligned portion of the reads from bam-file to SMRT-SV insertion sequences and kept only insertions with an average coverage of ≥20× and 85% of covered bases.

**Figure 4.**
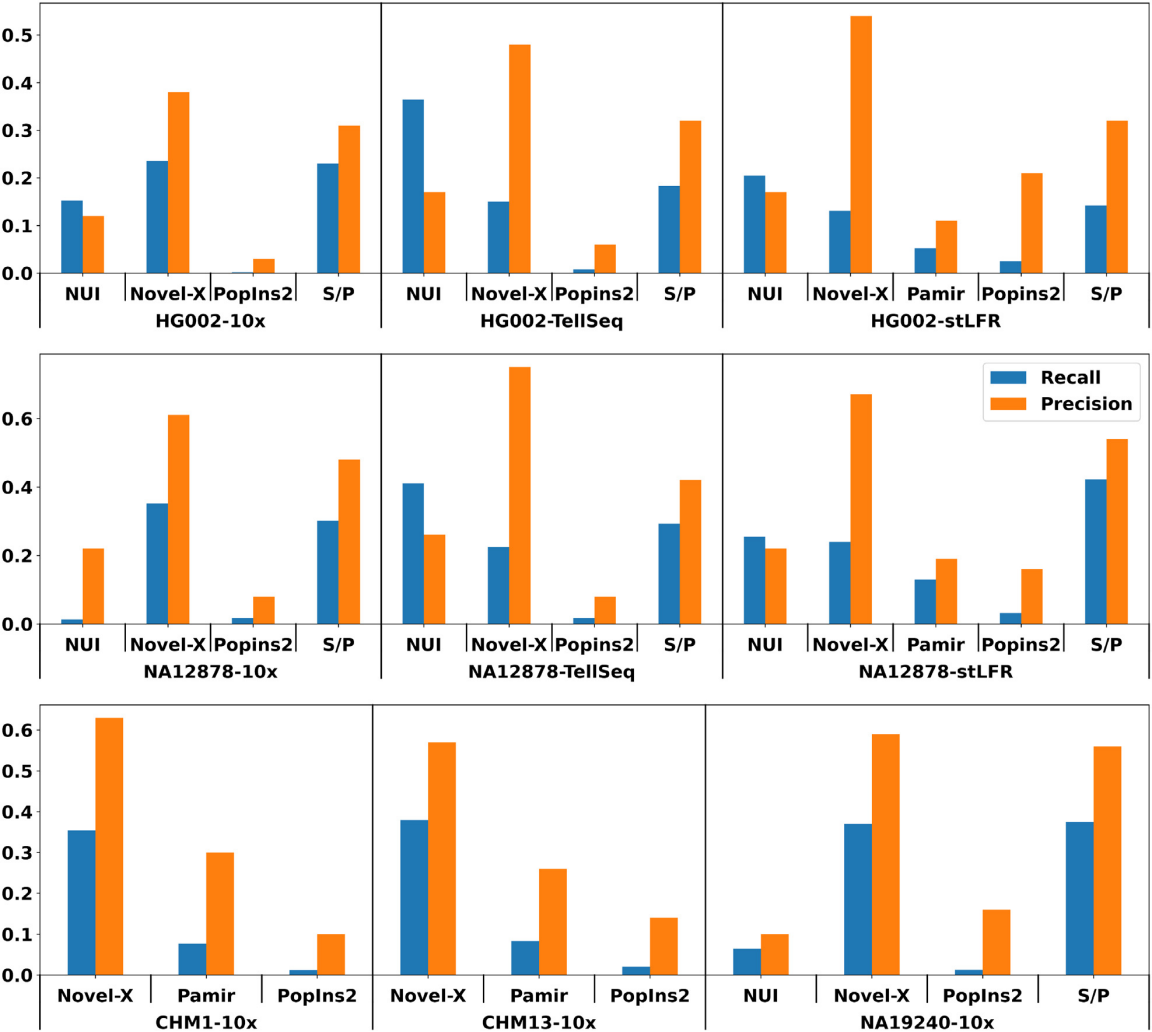
Benchmark overview. Results show that Novel-X consistently has higher precision regardless of the technology used. Supernova/Paftools and NUI have the highest recall on different datasets. For 10x datasets Novel-X performance is strictly better compared wit other tools, while Tell-Seq and stLFR datasets show example of a trade-off where Novel-X has the best precision but Supernova/Paftools and NUI have better recall on different datasets. S/P refers to SuperNova/Paftools.

The comparison results of different tools on the nine chosen datasets are presented in Figure 4. Our results show that Novel-X achieves better recall and precision on 10x datasets, except NA19240-10x. For the NA19240-10x dataset, compared with SuperNova/Paftools, Novel-X achieved a slightly better precision (59% versus 56%), but a slightly lower recall (37.0% versus 37.4%). For Tell-Seq and stLFR datasets, Novel-X achieved significantly higher precision compared with other tools, while SuperNova/Paftools and NUI-pipeline achieved a better recall. Here we conclude that Novel-X is the tool of choice if downstream application demands higher precision. Novel-X performs differently on different synthetic long-read technologies. The 10x platform results typically have a higher recall but a lower precision compared with Tell-Seq and stLFR platforms.

We present comparison results of different tools in more detail in Supplementary Table S3, which includes NRS insertion length breakdown. We were also interested in whether insertions that were called are repetitive or not. In order to test this hypothesis, we compared benchmark results against non-filtered validation sets. The comparison against non-filtered datasets is presented in Supplementary Table S4. These results show that the majority of insertions that were called by NUI-pipeline were not unique. Novel-X, Pamir, PopIns2 and SuperNova/Paftools also increased their precision up to 40% due to insertions that are repetitive. This fact hints that most of the unmatched called insertions are real, though repetitive. After we relaxed the NRS insertion length condition, we found that an additional 15–30% PopIns2 calls have correct insertion positions. However, the reported length of insertions and therefore content is usually incorrect, significantly lowering both the precision and recall in our benchmarks.

We also performed additional experiments on the CHM1-10x dataset that allowed us to orthogonally confirm our results and analyze sequence content around NRSs. We were interested in whether there were NRS locations inside repetitive regions. Novel insertion detection tools for the short-read data produce anchors that are limited by the short insertion size (e.g. only 300 bp for an anchor). Mapping such short sequences to repetitive regions is often a challenging task that cannot be resolved unambiguously, while longer anchor size can resolve this problem spanning through the repetitive region. In order to show that insertion sites are common inside repeats, we extracted the sequences of the regions 300 bp upstream and downstream of insert sites from the CHM1-10x dataset and searched these sequences for repeats using RepeatMasker (http://www.repeatmasker.org). In total, from the callset produced by Novel-X, 40 insertions were located in repetitive regions of the reference genome. We confirmed these results using UCSC mappability tracks. For k100 and k50 Umap Multitrack, we found 24 and 93 insertions in repetitive regions. PopIns2 and Pamir were able to call only one and three insertions identified with k100 Umap Multitrack, respectively.

As an alternative way of validating our CHM13 insertions, we compared the list of barcodes associated with every insertion call with all the barcodes aligned to the vicinity of the reported insertion breakpoint on the reference genome. For each insertion breakpoint, we extracted barcodes in a 10 kbp window around it. We found that the barcode lists for the majority of the insertions are completely or almost completely included in the corresponding barcode list for their insertion sites. This fact gives additional support that our insertions are correctly placed on the reference genome (see Supplementary Figure S2).

The CHM13 genome provides a unique opportunity to check how well Novel-X calls align with whole-genome assembly. We validated our Novel-X calls using the recent T2T consortium (45) assembly (v2.0) of the CHM13 genome. To obtain this high-quality assembly, the T2T Consortium used HiFi PacBio reads for the initial assembly and ONT Ultra-Long reads for the polishing step. We aligned this assembly to the GRCh38 reference genome using Minimap2, extracted long insertions from the vcf file and compared these insertions with Novel-X calls. We found that 78% of Novel-X calls are confirmed with the T2T assembly.

Finally, we also checked if Novel-X CHM1 NRSs overlap with known genes and their coding sequences. We compared our reported breakpoint positions on the reference with GENCODE annotation v.24 (46). A total of 111 out of 226 insertions fall inside known gene sequences, but only two of those fall into the exonic region of known protein-coding genes. In these data, it appears that NRS insertions may contain novel exons or important non-coding regions, but they do not necessarily disrupt known exonic sequences.

### Computational resource requirements

We measured Novel-X runtime and memory consumption on Intel Xeon Gold 6230 2.1-3.9GHz processors using 32 cores on the HG002 GIAB genome using Snakemake internal tools. Novel-X took 22 h and the peak memory usage was 246 Gb of RAM during assembly of unmapped reads. PopIns2 requires only 2 h 15 min to finish, with a peak memory of 119 Gb. While the Novel-X approach runs more slowly than existing short-read methods (e.g. PopIns2), it is able to find substantially more novel insertions confirmed with PacBio data. An alternative is to use whole-genome assembly (such as NUI or SuperNova/Paftools). However, whole-genome assembly requires significantly more computational resources than Novel-X. SuperNova assembly of the HG002 dataset itself with the same parameters requires 107 h with a peak memory of 373 Gb. Later steps of NUI and the SuperNova/Paftools pipeline require additional running time, though they are incomparably faster than genome assembly.

### Novel-X enables discovery of novel insertions in a diverse cohort

We ran our method on 68 whole-genome SLR datasets from diverse human populations. These samples are originally a part of the HapMap and 1000 Genomes Projects. Populations include Caucasian (1 sample), Finnish (1), Italy/Toscani (1), Southern Han Chinese (2), Han Chinese (8), Japanese (9), Puerto Rican (1), USA/Mexican (1), Utah/Mormon (20), USA/African (5) and Yoruba/Nigerian (19 samples). The samples were prepared on the 10x Genomics Chromium platform and Illumina sequenced on HiSeq 4000 systems as part of the Polaris PGx cohort. Note that they are also among the samples from the Centers for Disease Control and Prevention’s Genetic Testing Reference Materials Coordination Program (GeT-RM) and were previously characterized for clinically important mutations (47).

**Figure 5.**
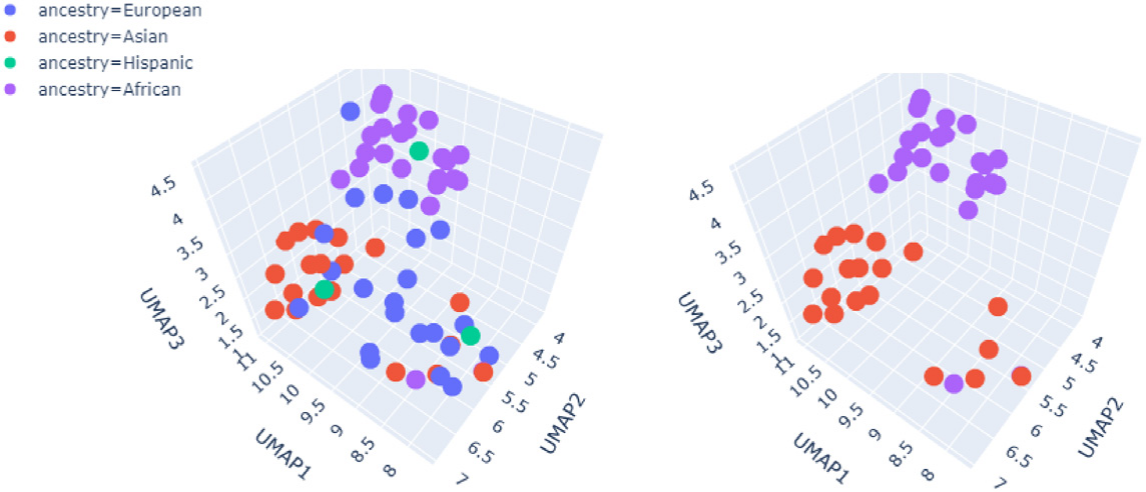
3D UMAP projection of insertion sites for each sample, colored by ancestry group. Left: all samples. Right: only samples from Asian and African ancestry groups. UMAP projection shows a clear separation between the two ancestry groups.

Novel-X found 16 691 insertion events larger than 300 bp across all genomes, with the longest insertion length of 85 908 bp and a total length of 18.2 Mb. Chromosome 2 contained the largest number of insertions (2749), followed by chromosome 1 with 2442 insertions. We removed short contigs of < 300 bp in length for downstream analysis and divided the genome into 10 kbp bins. Insertion spots falling into the same 10 kbp bins were identified as the same unique insertion site (UIS). Accordingly, across all samples, we have 16 691 insertions in total, distributed among 2405 UISs. An overview of population-specific UIS locations is shown in Figure 5. Samples were projected into 3D UMAP space based on their UISs. The panel on the left shows the projection of all samples colored by populations, while the panel on the right specifically compares African and Asian populations. Novel-X was able to identify population-specific insertions. For each insertion, we further calculated the percentage of samples in each ancestry in which the insertions occur. Insertions which occur in < 80% of samples in one ancestry group are filtered out. Then for each frequent insertion, we count the number of ancestries in which it arises. Insertions that occur in a specific ancestry group were marked as ancestry-unique insertions. For example, insertion on chromosome 4, in the 156 847 000–156 848 000 region occurred in most of the samples of African ancestry, but in none of the other samples. A list of 237 ancestry-unique insertions is available in Supplementary Table S5.

**Figure 6.**
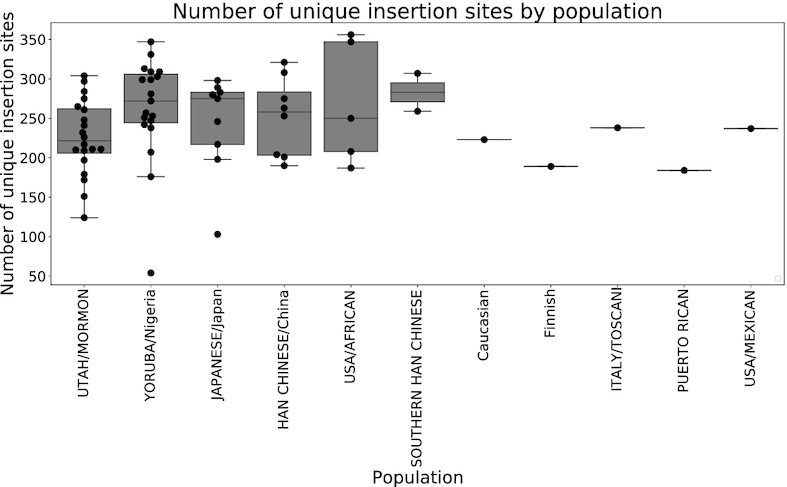
Distribution of numbers of unique insertion sites for each population. The *x*-axis denotes populations, while the *y*-axis shows the number of unique insertion sites for all samples in each population.

**Figure 7.**
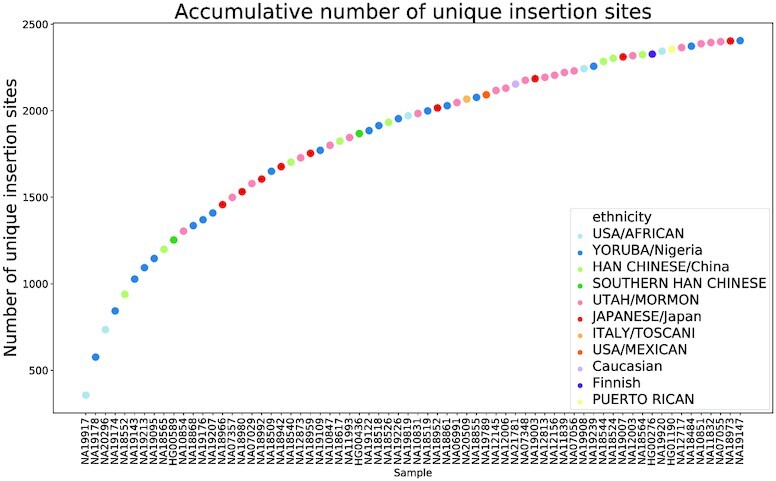
Accumulative number of unique insertion sites by including more samples. Samples are shown on the *x*-axis ranked by the descending order of number of unique insertion sites. Colors represent the population category of each sample.

The distribution of UIS counts for all samples in each population is shown in Figure 6. The Japanese population in our cohort has an average highest number of UISs, while the largest variance in the number of UISs was observed in the USA/African population. Different samples may share the same insertion sites, and an accumulative number of UISs identified by all samples is shown in Figure 7. Samples are ranked with a decreasing number of UISs. As more samples were included, the number of UISs observed plateaued.

Previously, we have shown that synthetic long reads allow us to place more contigs into the reference genome. Inserted contigs are identified by Novel-X, with the distribution of contig length shown in Supplementary Figure S3. Insertions in chromosomes 3, X and 21 show longer sequences, with an average length of 1641, 2021 and 3093 bp, respectively. Anchor length distribution is also shown in Supplementary Figure S3, with a median length of 1601 bp.

To further understand the biological effects of insertions, we annotated all insertion loci using SnpEff v4.3T (48), and checked whether the insertions overlap with coding regions and known genes. For each insertion, a putative impact was calculated to measure the level of disruption. There are four categories of impact: high, moderate, low and modifier. High impact refers to variants that have a disruptive impact on the protein, possibly causing protein truncation, or loss of function. The moderate effect refers to a non-disruptive variant that may change protein effectiveness. Low impact refers to variants that are mostly harmless, while modifiers refer to variants affecting non-coding genes or with no evidence for impact. As we expected, the majority of insertions would have only low to modifier effects. Among 7490 insertions which fall into the transcript region, 7238 of them have a modifier effect, while only 182 have a high impact. Among the genes located in the region with high impact insertions, 29 of them were predicted as frameshift or stop codons introduced by the insertion.

**Figure 8.**
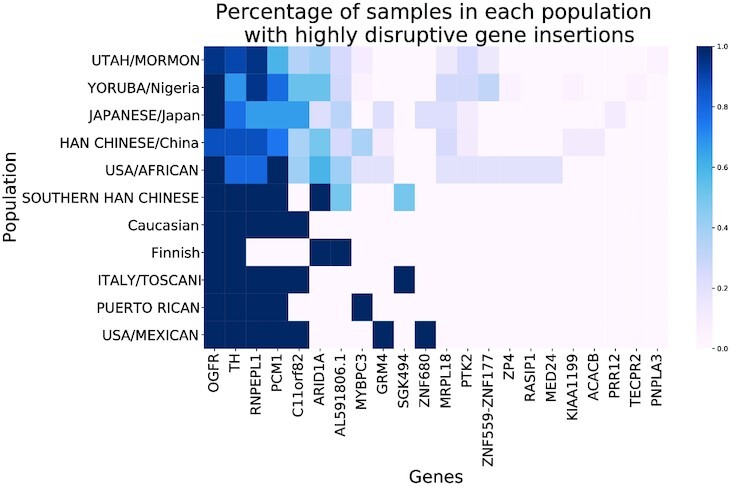
Fraction of samples in each population with highly disruptive gene insertions.

We also compared these highly disruptive insertions with insertion hotspots (see Figure 8; Supplementary Figure S4), and identified four highly disrupted genes overlapping with insertion hotspots as we have defined above (≥30 samples with insertion around the hotspot). They are ZP4 from Chr1, RNPEPL1 from Chr2, TH from Chr11 and OGFR from Chr20. As an example, in Supplementary Figure S5, we show the effect on RNPEPL1.

## DISCUSSION

We have described a novel strategy for assembly of multiple barcodes originating from the same genomic loci. This strategy has the potential to be used in the assembly of other classes of complex SVs and we are planning to explore this in future work. Such a strategy greatly decreases the complexity of genomic loci assembly even with a complex repeat composition because these repeats can be distinguished using the encoded barcode information.

One of the problems we faced during development of our method was the choice of an assembler. Most of the algorithms that use assembly-based techniques employ Velvet (34) as the assembler of choice. We also used Velvet for this study because it is a reliable and conservative choice. Note that SuperNova 2.1, a newer release of the original SuperNova software developed by a team at 10x Genomics (9), can also be an alternative choice. In the case of SuperNova assembler, however, the user has no control over any assembler parameters, and default parameters are maximally tuned for whole human genome diploid assembly. From our experience with SuperNova with default parameters, we believe that some viable novel insertion candidates were dropped during the assembly phase due to low coverage. A future direction would be to develop a specific local assembler designed for SV detection tasks in synthetic long-read data.

Note that, while our analysis focuses on insertions longer than 300 bp, our method can indeed detect and report smaller insertions too (i.e. in the 50–300 bp range). However, for such small insertions, we do not recommend Novel-X because the long-range information used in Novel-X would not be helpful. In order to successfully assemble a novel insertion, Novel-X needs a substantial number of distinct barcodes to be associated with it. For small insertions, the number of associated barcodes is usually small and the coverage is not sufficient for a successful anchor assembly. Given the current read length and short fragment sizes of standard Illumina sequencing, short-read techniques already have good performances for detecting these small events.

We applied our method to the Polaris HiSeq 4000 PGx 10x Genomics cohort and demonstrated that Novel-X enables large-scale discoveries. Our results can be used as a resource for the broader genomics community. We are planning to use Novel-X in other large-scale projects in the near future. There are already thousands of genomes sequenced on synthetic long-read platforms including a large cohort the InPSYght Consortium that can be screened using our method.

## DATA AVAILABILITY

The source code is freely available at https://github.com/1dayac/novel_insertions. The supplementary data including VCFs that were used for benchmarks are available at https://github.com/1dayac/novel_insertions_supplementary. Instructions on how to run the comparisons are available in Supplementary text B: ‘Supplementary repository’.

The Polaris HiSeq 4000 PGx 10x Genomics Cohort can be obtained from NCBI SRA (Accession: PRJEB26950 ID: 474329). A link to these data is also available at the Polaris project GitHub repository: https://github.com/Illumina/Polaris/wiki/HiSeqX-PGx-Cohort. The CHM1, CHM13, NA12878, NA19240 and HG002 genomes can be downloaded from https://support.10xgenomics.com/de-novo-assembly/datasets. CHM1 and CHM13 WGS Illumina data are available through ERX1413366 and ERX1413367 SRA accessions, respectively.

CHM1 and CHM13 callsets can be downloaded from http://eichlerlab.gs.washington.edu/publications/Huddleston2016/structural_variants/. The NA19240 callset can be download from http://www-rcf.usc.edu/∼mchaisso/hgsvg/CombinedVCFs/NA19240.sv_calls.vcf.gz. The NA12878 callset can be download from https://ftp-trace.ncbi.nlm.nih.gov/giab/ftp/data/NA12878/NA12878_PacBio_MtSinai/. The HG002 callset can be downloaded at ftp://ftp-trace.ncbi.nlm.nih.gov/giab/ftp/release/AshkenazimTrio/HG002_NA24385_son/NIST_SV_v0.6/. The CHM13 T2T assembly v2.0. used for CHM13 calls validation can be found at https://github.com/nanopore-wgs-consortium/chm13.

## Supplementary Material

gkac653_Supplemental_File
