# Supplementary material for: Efficient detection and assembly of non-reference DNA sequences with synthetic long reads
Source: Nucleic Acids Res. 2022 Aug 4;50(18):e108. doi: 10.1093/nar/gkac653 (PMC9561269; doi:10.1093/nar/gkac653)
Supplement: gkac653_Supplemental_File [file gkac653_supplemental_file.pdf]

Supplementary Data: Efficient detection and assembly of non-reference DNA sequences with synthetic long reads

Dmitry Meleshko[1,2], Rui Yang[1], Patrick Marks[3], Stephen Williams[3], and Iman Hajirasouliha[2,4,*]

[1]Tri-Institutional Computational Biology & Medicine Program, Weill Cornell Medicine of Cornell University, NY, 10021, USA
[2]Institute for Computational Biomedicine, Department of Physiology and Biophysics, Weill Cornell Medicine of Cornell University, NY, 10021, USA
[3]10x Genomics Inc., Pleasanton, California, 94566, USA
[4]Englander Institute for Precision Medicine, The Meyer Cancer Center, Weill Cornell Medicine, NY, 10021, USA
*Corresponding author

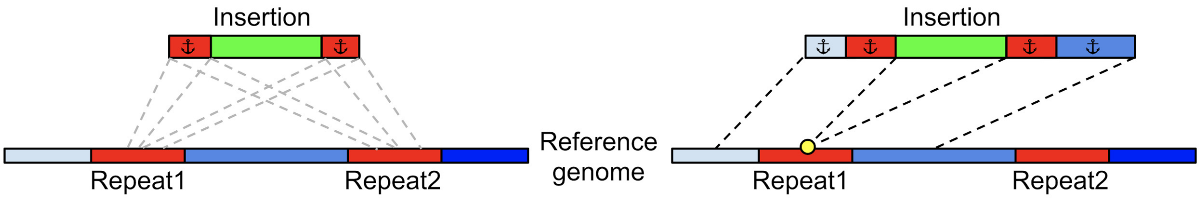

Figure S1: Schematic comparison of NRS detection algorithms with standard short-reads (left) and linked-reads (right). Shortcomings for existing short-read algorithms often arise from the fact that insertions can take place inside repetitive regions. Left: anchors constructed with paired-end data are too short to be uniquely placed on the reference genome as they can be mapped to any copy of repeat (shown in red). Right: linked-read sequencing data provides information that can be used for assembling long anchors that span repeat and the insertion breakpoint can be unambiguously placed on the reference genome.

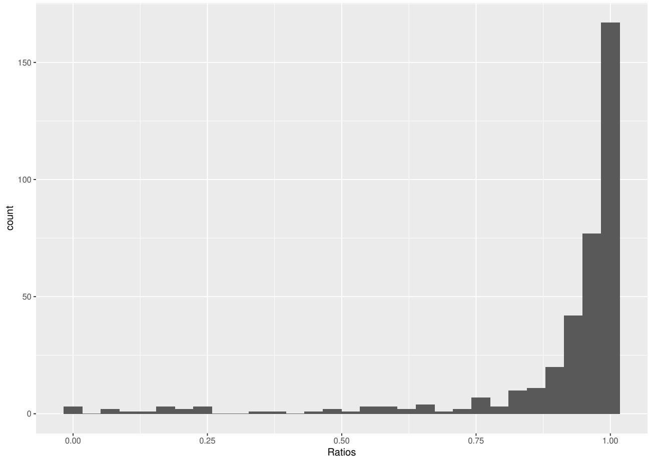

Figure S2: Histogram of intersection of barcodes associated with insertions and found on the reference to associated with insertion barcodes ratio.

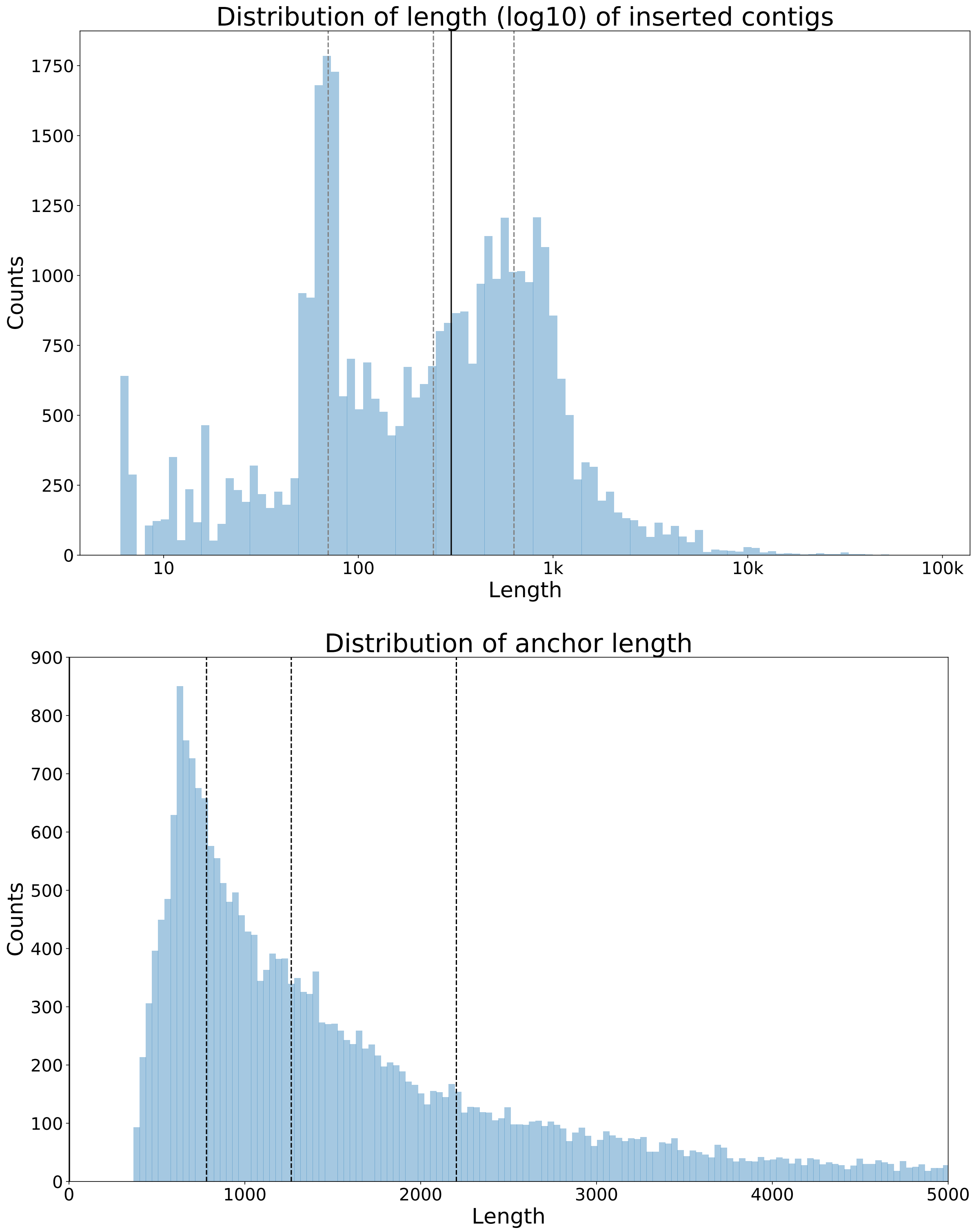

Figure S3: **NRS properties for 68 samples cohort. Top**: Distribution of length of inserted contigs in log10 scale across all populations. Black vertical line represents 300 bp cut-off threshold for short insertions. Dashed vertical lines represent (0.25, 0.5, 0.75) quartile of distribution, locate at (70, 243, 630) respectively. **Bottom**: Distribution of anchor length across all populations. Dashed vertical lines represent (0.25, 0.5, 0.75) quartile of distribution, locate at (852, 1601, 3667) respectively.

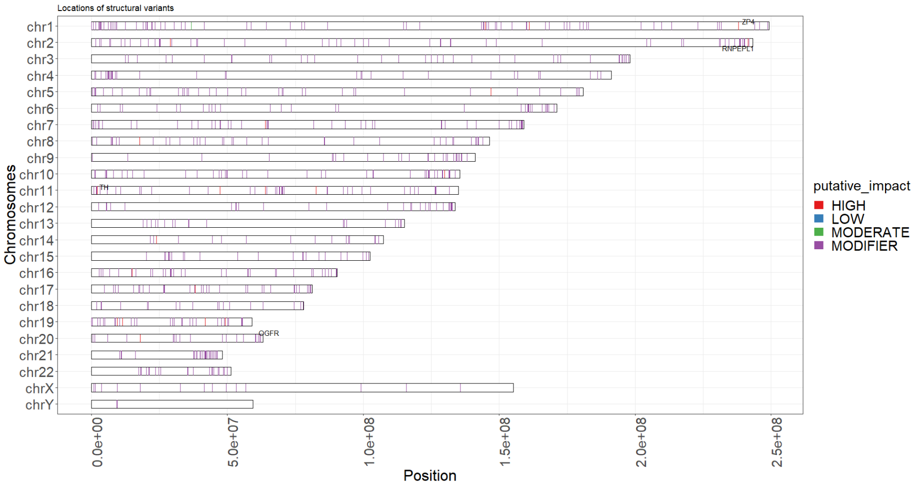

Figure S4: Chromatin location of insertion hotspots and highly disrupted genes. Vertical marks show insertion locations across chromosomes, colored by putative impact. Highly disrupted genes located at insertion hotspots (more than 30 samples have insertions) are annotated.

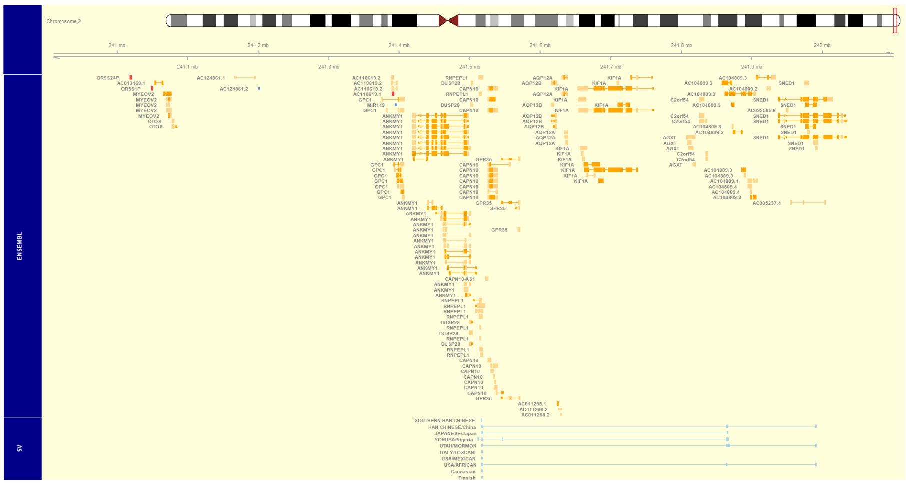

Figure S5: Overlapped region of insertion hotspot and highly disrupted gene RNPEPL1. Top track represents Chromosome 2 and corresponding location of overlap regions. ENSEMBL track represents genes located the region. Insertion track shows length and position of inserted contigs, linked within population.

Method	TP	FP	Missed	Sensitivity	Precision	F1
			Coverage = 13X			
Novel-X	763	2	1237	0.381	0.997	0.552
PopIns2	118	0	1882	0.06	1.0	0.11
NUI	1362	3	638	0.68	0.998	0.81
			Coverage = 26X			
Novel-X	1803	3	197	0.902	0.998	0.948
PopIns2	627	0	1373	0.31	1.0	0.477
NUI	1725	2	275	0.863	0.999	0.923
			Coverage = 39X			
Novel-X	1842	1	158	0.921	0.9995	0.959
PopIns2	1124	0	876	0.562	1.0	0.720
NUI	1754	2	246	0.88	0.999	0.934
			Coverage = 52X			
Novel-X	1855	1	145	0.928	0.999	0.962
PopIns2	1426	1	574	0.713	0.999	0.832
NUI	1746	3	254	0.873	0.998	0.931

Table S1: Comparison of Novel-X, PopIns2 and NUI-pipeline performance on downsampled data.

Assembly	Insertion reassembly	TP	Missed	FP
Velvet	SPAdes	1789	211	1
SPAdes	SPAdes	1680	320	5
Velvet	Supernova	1150	850	2
Supernova	SPAdes	1705	295	7
Velvet	Velvet	1386	614	0

Table S2: Performance of different assembly strategies on simulated data.

Novel insertions detection results for insertions longer than 300 bp						
Length (bp)	Validation set	Novel-X	Pamir	PopIns2	NUI	Supernova/ Paftools
**HG002 - 10x (59×)**						
300-499	133	99 (**42**, **42%**)	-	17 (1, 6%)	288 (24, 8%)	117 (36, 30%)
500-999	215	150 (**58**, **39%**)	-	10 (0, 0%)	156 (34, 22%)	159 (48, 30%)
1000-1999	103	48 (14, 29%)	-	2 (0, 0%)	89 (11, 12%)	64 (**20**, **31%**)
≥2000	62	18 (7, **39%**)	-	0 (0, 0%)	45 (9, 20%)	128 (**14**, 11%)
Total(≥300)	513	315 (**121**, **38%**)	-	29 (1, 3%)	661 (78, 12%)	382 (118, 31%)
Max length (bp)	24323	5690	-	747	7194	**10203**
**HG002 - UST Tell-Seq (42×)**						
300-499	133	68 (36, **75%**)	-	42 (2, 5%)	410 (**52**, 13%)	99 (29, 29%)
500-999	215	77 (34, **44%**)	-	21 (2, 10%)	308 (**84**, 27%)	141 (48, 34%)
1000-1999	103	11 (5, **45%**)	-	1 (0, 0%)	182 (**37**, 20%)	45 (14, 10%)
≥2000	62	3 (2, **67%**)	-	0 (0, 0%)	182 (**14**, 8%)	7 (3, 43%)
Total(≥300)	513	159 (77, **48%**)	-	64 (4, 6%)	1082 (**187**, 17%)	292 (94, 32%)
Max length (bp)	24323	3630	-	562	**13470**	3631
**HG002 - stLFR (35×)**						
300-499	133	45 (21, **47%**)	162 (19, 12%)	32 (7, 22%)	259 (**39**, 15%)	91 (32, 35%)
500-999	215	66 (39, **59%**)	73 (8, 11%)	27 (6, 22%)	170 (**44**, 26%)	96 (31, 32%)
1000-1999	103	9 (5, **56%**)	11 (0, 0%)	2 (0, 0%)	94 (**16**, 17%)	35 (8, 23%)
≥2000	62	3 (2, **67%**)	0 (0, 0%)	1 (0, 0%)	91 (**6**, 7%)	8 (2, 25%)
Total(≥300)	513	123 (67, **54%**)	246 (27, 11%)	62 (13, 21%)	614 (**105**, 17%)	230 (73, 32%)
Max length (bp)	24323	3714	963	958	**10549**	7360
**NA12878 - 10x (60×)**						
300-499	138	103 (**65**, **63%**)	-	19 (4, 21%)	390 (33, 8%)	92 (41, 44%)
500-999	198	120 (**73**, **61%**)	-	8 (2, 25%)	178 (45, 25%)	123 (58, 47%)
1000-1999	96	35 (**20**, **57%**)	-	0 (0, 0%)	109 (18, 17%)	41 (17, 41%)
≥2000	94	43 (27, **63%**)	-	4 (1, 25%)	177 (34, 19%)	74 (**42**, 57%)
Total(≥300)	526	301 (**185**, **61%**)	-	31 (7, 23%)	854 (130, 15%)	330 (158, 48%)
Max length (bp)	20442	**27836**	-	3410	12666	4935
**NA12878 - UST Tell-Seq (41×)**						
300-499	138	62 (44, **71%**)	-	55 (2, 4%)	330 (**62**, 19%)	131 (36, 27%)
500-999	198	58 (43, **74%**)	-	40 (4, 10%)	240 (**87**, 36%)	138 (60, 43%)
1000-1999	96	21 (17, **81%**)	-	9 (1, 11%)	123 (**33**, 27%)	55 (31, 56%)
≥2000	94	16 (14, 88%)	-	2 (2, **100%**)	128 (**34**, 27%)	41 27, 66%)
Total(≥300)	526	157 (118, **75%**)	-	106 (9, 8%)	821 (**216**, 26%)	367 (154, 42%)
Max length (bp)	20442	12143	-	4135	12667	**19696**
**NA12878 - stLFR (40×)**						
300-499	138	74 (50, **68%**)	232 (31, 13%)	59 (2, 3%)	262 (41, 16%)	128 (**68**, 53%)
500-999	198	65 (44, **68%**)	110 (31, 28%)	31 (8, 26%)	169 (52, 31%)	171 (**91**, 53%)
1000-1999	96	27 (18, **67%**)	21 (6, 29%)	10 (5, 50%)	78 (20, 26%)	70 (**35**, 50%)
≥2000	94	22 (14, **64%**)	2 (0, 0%)	5 (2, 40%)	87 (21, 24%)	45 (**28**, 62%)
Total(≥300)	526	188 (126, **67%**)	365 (68, 19%)	105 (17, 16%)	596 (134, 22%)	414 (**222**, 54%)
Max length (bp)	20442	12074	1961	4962	12147	**12667**
**CHM1 - 10x (40×)**						
300-499	101	73 (**41**, **56%**)	61 (13, 21%)	20 (2, 0%)	-	-
500-999	138	99 (**60**, **61%**)	34 (13, 38%)	20 (2, 10%)	-	-
1000-1999	85	33 (**23**, 70%)	6 (5, **83%**)	7 (1, 14%)	-	-
≥2000	77	21 (**18**, **86%**)	1 (0, 0%)	3 (2, 67%)	-	-
Total(≥300)	401	226 (**142**, **63%**)	102 (31, 30%)	50 (5, 10%)	-	-
Max length (bp)	27836	**10436**	2072	2893	-	-
**CHM13 - 10x (40×)**						
300-499	85	71 (**33**, **46%**)	68 (12, 18%)	25 (0, 0%)	-	-
500-999	126	100 (**53**, **53%**)	29 (7, 24%)	16 (4, 25%)	-	-
1000-1999	74	41 (**29**, 71%)	8 (6, **75%**)	6 (2, 33%)	-	-
≥2000	63	36 (**27**, 75%)	4 (4, **100%**)	2 (1, 50%)	-	-
Total(≥300)	348	248 (**142**, **57%**)	109 (29, 26%)	49 (7, 14%)	-	-
Max length (bp)	20444	**12146**	4136	2159	-	-
**NA19240 - 10x (73×)**						
300-499	167	118 (**57**, **48%**)	-	29 (3, 10%)	162 (8, 5%)	122 (53, 43%)
500-999	238	156 (**94**, 60%)	-	14 (2, 14%)	66 (13, 20%)	145 (90, **62%**)
1000-1999	109	53 (32, **60%**)	-	5 (1, 20%)	76 (13, 17%)	64 (**35**, 55%)
≥2000	113	68 (49, **72%**)	-	3 (2, 67%)	77 (8, 10%)	86 (**57**, 66%)
Total(≥300)	627	395 (232, **59%**)	-	51 (8, 16%)	381 (40, 10%)	417 (**235**, 56%)
Max length (bp)	27821	**27836**	-	10228	14919	19815

Table S3: Length breakdown and comparison between the validation set (constructed using SMRT-SV callset or multiple orthogonal methods callset), short-read methods Pamir and PopIns2, and linked-read methods Novel-X, NUI, and Supernova/Paftools for 10x Genomics datasets. The numbers in brackets indicate the count of overlaps with SMRT-SV calls and the percentage of the overlapping calls. As it can be seen, the number of validated novel insertion calls using our method is significantly higher than those obtained by short-read methods.

Novel insertions detection results for insertions longer than 300 bp						
Length (bp)	Validation set	Novel-X	Pamir	PopIns2	NUI	Supernova/ Paftools
**HG002 - 10x (59×)**						
300-499	2771	99 (75, 76%)	-	17 (3, 17%)	288 (**230**, **80%**)	99 (57, 58%)
500-999	1670	150 (107, 71%)	-	10 (4, 40%)	156 (**120**, **77%**)	141 (101, 72%)
1000-1999	1070	48 (27, 56%)	-	2 (1, 50%)	89 (**71**, **80%**)	45 (27, 60%)
≥2000	1111	18 (15, **83%**)	-	0 (0, 0%)	128 (**102**, 80%)	7 (6, 86%)
Total(≥300)	6622	315 (224, 71%)	-	29 (8, 28%)	661 (**523**, **79%**)	292 (191, 65%)
Max length (bp)	125188	5690	-	1024	**27836**	3631
**HG002 - UST Tell-Seq (42×)**						
300-499	2771	68 (58, **85%**)	-	42 (8, 19%)	410 (**337**, 82%)	117 (46, 39%)
500-999	1670	77 (66, **86%**)	-	21 (4, 19%)	308 (**217**, 70%)	159 (89, 56%)
1000-1999	1070	11 (9, 82%)	-	1 (1, **100%**)	182 (**133**, 73%)	64 (37, 58%)
≥2000	1111	3 (3, **100%**)	-	0 (0, 0%)	182 (**149**, 82%)	42 (30, 71%)
Total(≥300)	6622	159 (136, **85%**)	-	64 (13, 20%)	1082 (**836**, 77%)	382 (202, 53%)
Max length (bp)	125188	3630	-	1024	**13470**	10203
**HG002 - stLFR (35×)**						
300-499	2771	45 (38, **84%**)	162 (29, 18%)	32 (4, 13%)	259 (**212**, 82%)	91 (55, 60%)
500-999	1670	66 (60, **91%**)	73 (11, 15%)	27 (8, 30%)	170 (**118**, 69%)	98 (66, 67%)
1000-1999	1070	9 (7, **78%**)	11 (0, 0%)	2 (0, 0%)	94 (**71**, 76%)	35 (14, 40%)
≥2000	1111	3 (3, **100%**)	0 (0, 0%)	1 (0, 0%)	91 (**74**, 81%)	8 (3, 38%)
Total(≥300)	6622	123 (108, **88%**)	246 (40, 16%)	62 (12, 19%)	614 (**475**, 77%)	232 (138, 59%)
Max length (bp)	125188	3714	963	958	**10549**	7360
**NA12878 - 10x (60×)**						
300-499	3409	103 (78, **76%**)	-	19 (4, 21%)	390 (**282**, 72%)	92 (66, 72%)
500-999	1481	120 (86, 72%)	-	8 (3, 38%)	178 (**139**, 78%)	123 (102, **83%**)
1000-1999	848	35 (24, 69%)	-	0 (0, 0%)	109 (**95**, 87%)	41 (36, **88%**)
≥2000	1064	43 (32, 74%)	-	4 (1, 25%)	177 (**124**, 70%)	74 (**64**, **86%**)
Total(≥300)	6802	301 (220, 73%)	-	31 (8, 26%)	854 (**640**, 75%)	330 (268, **81%**)
Max length (bp)	28156	**27836**	-	3410	14920	15198
**NA12878 - UST Tell-Seq (41×)**						
300-499	3409	62 (52, **84%**)	-	55 (3, 5%)	330 (**262**, 79%)	101 (70, 69%)
500-999	1481	58 (47, **81%**)	-	40 (5, 13%)	240 (**171**, 71%)	128 (98, 77%)
1000-1999	848	21 (18, **86%**)	-	9 (1, 11%)	123 (**92**, 75%)	54 (40, 74%)
≥2000	1064	16 (14, 88%)	-	2 (2, **100%**)	128 (**102**, 80%)	38 (27, 71%)
Total(≥300)	6802	157 (131, **83%**)	-	106 (11, 10%)	821 (**627**, 76%)	321 (235, 73%)
Max length (bp)	28156	12143	-	4135	12667	**19696**
**NA12878 - stLFR (40×)**						
300-499	3409	74 (59, **80%**)	232 (45, 19%)	59 (4, 7%)	412 (**318**, 77%)	130 (93, 72%)
500-999	1481	65 (56, **86%**)	110 (33, 30%)	31 (8, 26%)	288 (**211**, 73%)	177 (131, 74%)
1000-1999	848	27 (20, 74%)	21 (6, 29%)	10 (5, 50%)	125 (**97**, **78%**)	73 (45, 62%)
≥2000	1064	22 (17, **77%**)	2 (0, 0%)	5 (2, 40%)	141 (**107**, 76%)	46 (31, 67%)
Total(≥300)	6802	188 (152, **81%**)	365 (84, 23%)	105 (19, 18%)	966 (**733**, 76%)	426 (300, 70%)
Max length (bp)	28156	12074	1961	4962	**12666**	**12667**
**CHM1 - 10x (40×)**						
300-499	3097	73 (**65**, **89%**)	61 (34, 56%)	20 (8, 40%)	-	-
500-999	1470	99 (**82**, **83%**)	34 (27, 79%)	20 (6, 30%)	-	-
1000-1999	852	33 (**26**, 79%)	6 (6, **100%**)	7 (2, 29%)	-	-
≥2000	981	21 (**19**, **90%**)	1 (0, 0%)	3 (2, 67%)	-	-
Total(≥300)	6400	226 (**192**, **85%**)	102 (67, 66%)	50 (18, 36%)	-	-
Max length (bp)	27836	**10436**	2072	2893	-	-
**CHM13 - 10x (40×)**						
300-499	3162	71 (**57**, **80%**)	68 (33, 49%)	25 (4, 16%)	-	-
500-999	1448	100 (**83**, **83%**)	29 (21, 72%)	16 (6, 38%)	-	-
1000-1999	851	41 (**34**, 83%)	8 (8, **100%**)	6 (2, 33%)	-	-
≥2000	875	36 (**32**, 89%)	4 (4, **100%**)	2 (1, 50%)	-	-
Total(≥300)	6336	248 (**206**, **83%**)	109 (66, 61%)	49 (13, 27%)	-	-
Max length (bp)	23307	**12146**	4136	2159	-	-
**NA19240 - 10x (73×)**						
300-499	4121	118 (93, **79%**)	-	29 (10, 34%)	162 (**125**, 77%)	122 (55, 45%)
500-999	1666	156 (**117**, 75%)	-	14 (3, 21%)	66 (54, **82%**)	145 (98, 68%)
1000-1999	1168	53 (38, 72%)	-	5 (1, 20%)	76 (**62**, **82%**)	64 (41, 64%)
≥2000	2531	68 (54, 80%)	-	3 (2, 67%)	77 (60, 78%)	86 (**66**, 77%)
Total(≥300)	8318	395 (**302**, 76%)	-	51 (16, 31%)	381 (301, **79%**)	417 (260, 62%)
Max length (bp)	79482	**27837**	-	10228	14919	19815

Table S4: Copy of Table S3 with comparison is performed against non-filtered SMRT-SV callset or multiple orthogonal methods callset.

Ancestry	Location	Samples with insertion	Total samples	Percentage
African	chr20 11312665	22	24	0.9167
African	chr16 71384688	20	24	0.8333
African	chr10 131512438	23	24	0.9583
European	chr20 61444279	21	22	0.9545
European	chr21 38914031	19	22	0.8636
European	chr2 218082962	22	22	1.0
European	chr2 234514767	22	22	1.0
European	chr2 236887596	20	22	0.9091
European	chr2 241517117	20	22	0.9091
European	chr2 2750405	22	22	1.0
European	chr3 126539876	18	22	0.8182
European	chr4 138867027	19	22	0.8636
European	chr4 38734186	19	22	0.8636
European	chr4 38754378	19	22	0.8636
European	chr4 7131367	22	22	1.0
European	chr6 11491594	18	22	0.8182
European	chr6 116047605	18	22	0.8182
European	chr6 165678196	18	22	0.8182
European	chr6 2376247	20	22	0.9091
European	chr6 39845370	21	22	0.9545
European	chr7 131654753	20	22	0.9091
European	chr8 142432223	21	22	0.9545
European	chr8 142502428	20	22	0.9091
European	chr8 21766795	21	22	0.9545
European	chr8 40820766	20	22	0.9091
European	chr8 47764037	19	22	0.8636
European	chr8 48174023	21	22	0.9545
European	chr9 121895762	19	22	0.8636
European	chr20 25067132	18	22	0.8182
European	chr20 22471994	19	22	0.8636
European	chr1 204195225	18	22	0.8182
European	chr9 123347685	22	22	1.0
Asian	chr9 88974428	17	19	0.8947
Asian	chrX 140723716	16	19	0.8421
European	chr10 131512438	19	22	0.8636
European	chr10 46526439	19	22	0.8636
European	chr10 49742424	21	22	0.9545
European	chr10 84601962	18	22	0.8182
European	chr11 126799470	22	22	1.0
European	chr11 2187926	20	22	0.9091
European	chr12 110880724	18	22	0.8182
European	chr12 57191929	20	22	0.9091
European	chr13 111435138	20	22	0.9091
European	chr13 111621629	19	22	0.8636
European	chr1 27086496	19	22	0.8636
European	chr13 112972801	19	22	0.8636
European	chr14 82287525	19	22	0.8636
European	chr15 26893428	21	22	0.9545
European	chr16 10040003	21	22	0.9545

European	chr16 87502298	20	22	0.9091
European	chr18 37283950	18	22	0.8182
European	chr18 63103455	19	22	0.8636
European	chr18 79541908	20	22	0.9091
European	chr19 1550008	18	22	0.8182
European	chr19 33250333	22	22	1.0
European	chr19 33458810	19	22	0.8636
European	chr1 168395496	19	22	0.8636
European	chr1 182138477	18	22	0.8182
European	chr13 22679414	18	22	0.8182
European	chr9 36764431	20	22	0.9091
European	chr9 88974428	21	22	0.9545
European	chr9 89254524	18	22	0.8182
Hispanic	chr21 41672810	3	3	1.0
Hispanic	chr21 7915747	3	3	1.0
Hispanic	chr2 218082962	3	3	1.0
Hispanic	chr2 236887598	3	3	1.0
Hispanic	chr2 241517117	3	3	1.0
Hispanic	chr2 2750405	3	3	1.0
Hispanic	chr3 72211325	3	3	1.0
Hispanic	chr4 101164092	3	3	1.0
Hispanic	chr4 38754378	3	3	1.0
Hispanic	chr4 7131367	3	3	1.0
Hispanic	chr5 135778794	3	3	1.0
Hispanic	chr6 11491592	3	3	1.0
Hispanic	chr6 116047604	3	3	1.0
Hispanic	chr6 167686999	3	3	1.0
Hispanic	chr6 3776803	3	3	1.0
Hispanic	chr7 102932561	3	3	1.0
Hispanic	chr7 156101312	3	3	1.0
Hispanic	chr7 31657167	3	3	1.0
Hispanic	chr8 130897691	3	3	1.0
Hispanic	chr8 139157950	3	3	1.0
Hispanic	chr8 142432223	3	3	1.0
Hispanic	chr8 142502431	3	3	1.0
Hispanic	chr8 17796330	3	3	1.0
Hispanic	chr8 41900622	3	3	1.0
Hispanic	chr8 47764037	3	3	1.0
Hispanic	chr21 38914020	3	3	1.0
Hispanic	chr21 38059653	3	3	1.0
Hispanic	chr21 37707714	3	3	1.0
Hispanic	chr20 61444268	3	3	1.0
European	chr9 93618898	18	22	0.8182
European	chrX 153382567	21	22	0.9545
Hispanic	chr10 131512438	3	3	1.0
Hispanic	chr11 126799421	3	3	1.0
Hispanic	chr11 2187926	3	3	1.0
Hispanic	chr11 30928615	3	3	1.0
Hispanic	chr11 70803384	3	3	1.0
Hispanic	chr12 132166674	3	3	1.0
Hispanic	chr12 132742762	3	3	1.0

Hispanic	chr13 111435138	3	3	1.0
Hispanic	chr13 112972799	3	3	1.0
Hispanic	chr14 100526933	3	3	1.0
Asian	chr9 123347685	18	19	0.9474
Hispanic	chr14 34047463	3	3	1.0
Hispanic	chr16 86013934	3	3	1.0
Hispanic	chr16 87502298	3	3	1.0
Hispanic	chr18 13982054	3	3	1.0
Hispanic	chr18 854878	3	3	1.0
Hispanic	chr19 29010235	3	3	1.0
Hispanic	chr19 33250333	3	3	1.0
Hispanic	chr19 33458810	3	3	1.0
Hispanic	chr1 168395496	3	3	1.0
Hispanic	chr1 230332053	3	3	1.0
Hispanic	chr1 25529503	3	3	1.0
Hispanic	chr1 34541476	3	3	1.0
Hispanic	chr20 61337292	3	3	1.0
Hispanic	chr14 82287525	3	3	1.0
Asian	chr9 121895747	16	19	0.8421
Asian	chr8 48174023	17	19	0.8947
Asian	chr8 47764037	16	19	0.8421
African	chr2 2750405	24	24	1.0
African	chr2 98660051	23	24	0.9583
African	chr3 126539876	23	24	0.9583
African	chr4 101164092	20	24	0.8333
African	chr4 138867044	22	24	0.9167
African	chr4 156847734	20	24	0.8333
African	chr4 157982654	22	24	0.9167
African	chr4 38734186	21	24	0.875
African	chr4 7131367	23	24	0.9583
African	chr5 42089532	21	24	0.875
African	chr6 167686999	20	24	0.8333
African	chr6 2376247	21	24	0.875
African	chr6 39845371	20	24	0.8333
African	chr8 1337419	20	24	0.8333
African	chr8 142502498	22	24	0.9167
African	chr8 17796249	20	24	0.8333
African	chr8 21766795	21	24	0.875
African	chr8 29820462	21	24	0.875
African	chr8 40820766	23	24	0.9583
African	chr8 47764037	22	24	0.9167
African	chr9 121895747	20	24	0.8333
African	chr9 123347685	22	24	0.9167
African	chr9 36764431	20	24	0.8333
African	chr9 88974428	22	24	0.9167
African	chrX 140723716	22	24	0.9167
African	chr2 241517117	22	24	0.9167
African	chr2 236887598	23	24	0.9583
African	chr2 235169315	21	24	0.875
African	chr2 234514767	23	24	0.9583
African	chr10 46526439	23	24	0.9583

African	chr10 49742424	20	24	0.8333
African	chr10 63783076	20	24	0.8333
African	chr10 84601964	20	24	0.8333
African	chr11 132125738	21	24	0.875
African	chr12 57191936	22	24	0.9167
African	chr13 111621629	21	24	0.875
African	chr13 112972801	21	24	0.875
African	chr13 22679414	21	24	0.875
African	chr15 26893415	23	24	0.9583
African	chr15 83695234	22	24	0.9167
African	chr15 94051934	22	24	0.9167
African	chrX 153382567	21	24	0.875
African	chr17 5622802	20	24	0.8333
African	chr18 63103455	20	24	0.8333
African	chr18 79541908	20	24	0.8333
African	chr19 29010235	20	24	0.8333
African	chr19 33250333	21	24	0.875
African	chr1 14492689	22	24	0.9167
African	chr1 168395498	22	24	0.9167
African	chr1 182138477	20	24	0.8333
African	chr20 22471994	22	24	0.9167
African	chr20 25067132	21	24	0.875
African	chr20 61444279	24	24	1.0
African	chr21 7915747	20	24	0.8333
African	chr2 218082962	20	24	0.8333
African	chr18 61644856	22	24	0.9167
Hispanic	chr8 48174023	3	3	1.0
Asian	chr10 63783073	16	19	0.8421
Asian	chr11 132125738	17	19	0.8947
Asian	chr2 218082962	17	19	0.8947
Asian	chr2 234514767	19	19	1.0
Asian	chr2 236887598	18	19	0.9474
Asian	chr2 241517117	16	19	0.8421
Asian	chr2 2750405	19	19	1.0
Asian	chr3 126539876	16	19	0.8421
Asian	chr3 72211314	16	19	0.8421
Asian	chr3 85548236	16	19	0.8421
Asian	chr4 157982644	17	19	0.8947
Asian	chr4 38734186	19	19	1.0
Asian	chr4 38754378	18	19	0.9474
Asian	chr4 7131367	19	19	1.0
Asian	chr6 11491594	16	19	0.8421
Asian	chr6 116047605	17	19	0.8947
Asian	chr6 157238333	16	19	0.8421
Asian	chr6 2376247	18	19	0.9474
Asian	chr6 39845370	19	19	1.0
Asian	chr7 102932561	16	19	0.8421
Asian	chr7 31657167	17	19	0.8947
Asian	chr7 99219897	16	19	0.8421
Asian	chr8 142432223	19	19	1.0
Asian	chr8 142502470	18	19	0.9474

Asian	chr8 21766795	17	19	0.8947
Asian	chr8 29820462	18	19	0.9474
Asian	chr8 40820766	19	19	1.0
Asian	chr2 14274479	17	19	0.8947
Asian	chr22 49276647	17	19	0.8947
Asian	chr21 7915747	17	19	0.8947
Asian	chr21 38508035	17	19	0.8947
Asian	chr11 2187926	16	19	0.8421
Asian	chr11 69291997	17	19	0.8947
Asian	chr12 110880724	16	19	0.8421
Asian	chr12 57191929	17	19	0.8947
Asian	chr13 111435138	17	19	0.8947
Asian	chr13 111621629	16	19	0.8421
Asian	chr13 112972801	18	19	0.9474
Asian	chr13 22679414	16	19	0.8421
Asian	chr15 26893428	17	19	0.8947
Asian	chr15 94051934	16	19	0.8421
Asian	chr16 10040003	17	19	0.8947
Asian	chr17 5622822	17	19	0.8947
Asian	chr10 84601964	17	19	0.8947
Asian	chr18 13982054	17	19	0.8947
Asian	chr18 79541908	17	19	0.8947
Asian	chr18 854878	18	19	0.9474
Asian	chr19 1550008	17	19	0.8947
Asian	chr19 33250333	19	19	1.0
Asian	chr19 33458810	17	19	0.8947
Asian	chr1 14492712	18	19	0.9474
Asian	chr1 168395498	18	19	0.9474
Asian	chr20 25067142	18	19	0.9474
Asian	chr20 25601604	18	19	0.9474
Asian	chr20 44404780	18	19	0.9474
Asian	chr20 58520304	16	19	0.8421
Asian	chr20 61444279	18	19	0.9474
Asian	chr18 63103470	16	19	0.8421
Hispanic	chr9 36764431	3	3	1.0

Table S5: List of ancestry specific novel sequence insertions. Last column shows the percentage of samples of a given population with this insertion.

Supplementary text A: "Proof: A random region has a low coverage"

Proof: We divide the genome into non-overlapping regions of size 100 kbp (an upper bound for a long molecule length). Thus, in a haploid human genome we get approximately 30,000 regions. Assuming a typical synthetic long read experiment provides 50× coverage, the number of different barcodes that are recruited by a single NRS insertion of would be close to 50. However, in a worst-case scenario we use at most 500 barcodes for the assembly. On average, 10 long fragments share a single barcode in typical 10x Genomics experiment, and for other technologies number of distinct barcodes is higher, therefore even less fragments share the same barcode. Given the 50× average coverage and at most 10 associated genomic fragments on average, we get

no more than 5000 underlying long fragments after we pool all reads with these barcodes. Let us assume that one long fragment falls exactly to one bin. Given this simplification, we can find the probability of getting 0,1,2,... pooled long fragments in a given off-target bin combinatorially. We can then write a formula

$$Pr(n) = \binom{N}{n} \frac{(B-1)^{N-n}}{B^N}$$

where B is a number of regions, N - number of long fragments and n - number of long fragments falling in a given bin. For the given values $Pr(n)$ is equal to $0.8465, 0.1411, 0.0118, 0.0007, 3 * 10^{-5}, \ldots$ for $n = 0,1,2,3,4\ldots$ in a worst-case scenario. Since each individual underlying genomic fragment is only fractionally covered by reads (0.1-0.2X), any region other than a region with true NRS sequence should not originate enough genomic fragments to have enough coverage for assembly.

Supplementary text B: "Supplementary repository"

Supplementary repository is available at https://github.com/1dayac/novel_insertions_supplementary. VCFs are stored in results folder. Subfolders correspond to different datasets we used - simulated, NA19240, HG002, HG002_stlfr, HG002_tellseq, NA12878, NA12878_stlfr, NA12878_tellseq, CHM1, CHM13, simulated. Simulated folder also contains VCFs for downsample experiment and different assemblers experiment. To evaluated VCFs we use compare_vcf.py script with dataset parameter.
